# Self‐Driving Microscopes: AI Meets Super‐Resolution Microscopy

**DOI:** 10.1002/smtd.202401757

**Published:** 2025-01-10

**Authors:** Edward N. Ward, Anna Scheeder, Max Barysevich, Clemens F. Kaminski

**Affiliations:** ^1^ Dept. Chemical Engineering and Biotechnology University of Cambridge Cambridge CB3 0AS UK

**Keywords:** deep learning, machine learning, microscopy, super‐resolution

## Abstract

The integration of Machine Learning (ML) with super‐resolution microscopy represents a transformative advancement in biomedical research. Recent advances in ML, particularly deep learning (DL), have significantly enhanced image processing tasks, such as denoising and reconstruction. This review explores the growing potential of automation in super‐resolution microscopy, focusing on how DL can enable autonomous imaging tasks. Overcoming the challenges of automation, particularly in adapting to dynamic biological processes and minimizing manual intervention, is crucial for the future of microscopy. Whilst still in its infancy, automation in super‐resolution can revolutionize drug discovery and disease phenotyping leading to similar breakthroughs as have been recognized in this year's Nobel Prizes for Physics and Chemistry.

## Graphical abstract

This review examines the use of machine learning to automate super‐resolution optical microscopy, enabling the microscope to autonomously make decisions on what, when, and how to image. By eliminating the need for human intervention, this approach has the potential to enhance the versatility and accessibility of super‐resolution microscopy. These advancements create new opportunities for investigating disease and accelerating drug discovery.

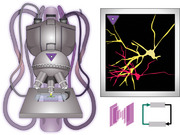

## 1 Introduction

**Figure 1:**
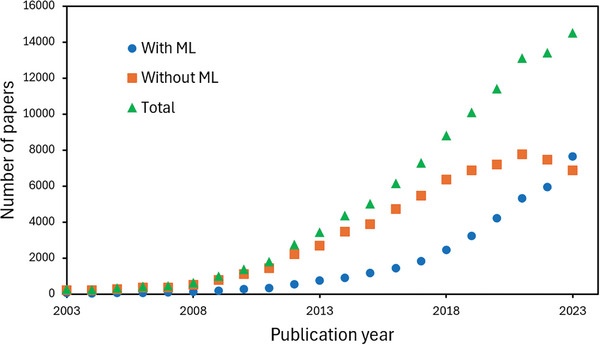
The use of ML in super‐resolution workflows is now commonplace. Green line: The number of publications cited in the Google Scholar database tagged with the keywords “super‐resolution” and “microscopy”. Orange line: Number of publications without ML, as determined by the number of publications tagged with “super‐resolution” and “microscopy” that did not contain any of the tags “deep learning”, “machine learning”, “reinforcement learning” or “AI”. Blue line: Number of publications making use of ML, calculated through the difference between green and yellow data points.

Modern fluorescence microscopy, including super‐resolution imaging, has been significantly enhanced by the advent of machine learning (ML). ML, and specifically deep learning (DL), represents a paradigm shift in computing. Through a capability to identify complex patterns in training data, DL can learn to approximate a task without the need to define a method, or even when such methods may not exist. This has countless applications in biological imaging, where the precise behaviors or morphologies of studied systems are rarely known or predictable. A drive toward open‐source code and availability of well‐documented libraries^[^ ^1^, ^2^ ^]^ has lowered the entry level for researchers from all backgrounds wishing to apply DL to their own applications. Even those unfamiliar with programming can now make use of the growing number of out‐of‐the‐box tools such as DeepImageJ,^[^ ^3^ ^]^ ZeroCostDL4Mic,^[^ ^4^ ^]^ and MMV_Im2IM,^[^ ^5^ ^]^ among others. This has resulted in a second renaissance in microscopy, and many of the latest developments have centered not around new hardware or techniques, but around the application of DL to existing technologies.^[^ ^6^, ^7^, ^8^, ^9^ ^]^ In super‐resolution microscopy specifically, an analysis of literature published over the past twenty years reveals a rapid increase of DL in the field. 2023 marked the first year when more work in super‐resolution microscopy made use of DL than did not (**Figure** **1**).

The vast majority of published work concerns the use of DL for post‐acquisition image processing tasks such as the denoising of images, fast reconstruction of image data, and the identification and classification of image objects.^[^ ^6^, ^7^, ^10^ ^]^

**Figure 2:**
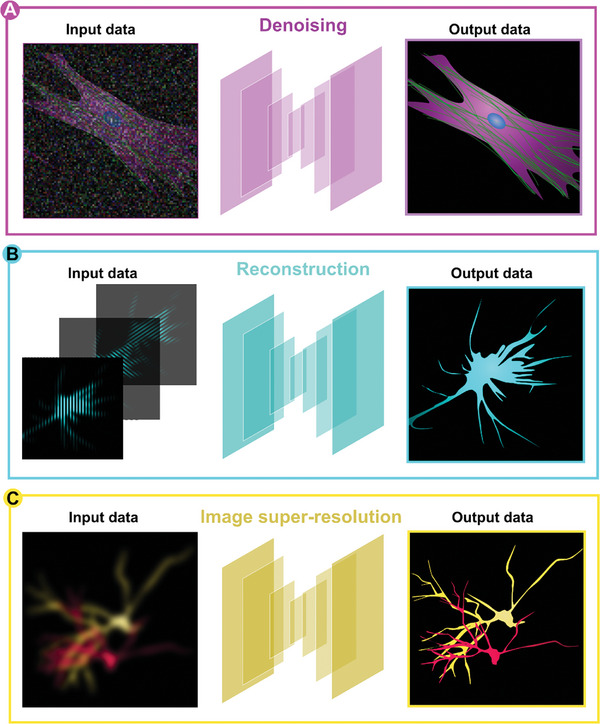
Applications of DL for the processing of super‐resolution image data. A) For image denoising tasks, low signal‐to‐noise ratio images are transformed to high signal‐to‐noise outputs. DL networks for denoising can either be trained to reconstruct specific targets or a range of samples. Denoising can also be performed either before or after additional image processing tasks such as deconvolution or super‐resolution reconstruction. B) DL networks for reconstruction take one or more images from a sequence and output a single super‐resolved image. By training DL networks on image data with noise, it is possible to incorporate denoising into the reconstruction process, and this has proven particularly effective in SMLM and SIM. C) DL networks are able to infer super‐resolution information directly from diffraction‐limited images. A challenge is the provision of suitable ground truth data on which the networks can be trained. This requires a priori knowledge of the sample, which is often not available.

Image denoising was one of the first applications of DL in microscopy (**Figure** **2**).^[^ ^11^ ^]^ Tools such as content aware image restoration (CARE)^[^ ^12^ ^]^ and Noise2Void^[^ ^13^ ^]^ pioneered this field, and DL methods are now the state‐of‐the‐art for denoising tasks.^[^ ^11^ ^]^ These approaches have also found their way into super‐resolution microscopy, for example stimulated emission depletion (STED) microscopy^[^ ^14^ ^]^ and structured illumination microscopy (SIM),^[^ ^15^, ^16^ ^]^ allowing the visualization of previously unobservable dynamic phenomena inside living cells.

In image reconstruction, DL can either wholly replace^[^ ^17^ ^]^ or supplement^[^ ^18^, ^19^ ^]^ classical algorithms. For SIM, this can improve both reconstruction fidelity and speed. Likewise, in single molecule localization microscopy (SMLM), DL offers a faster approach to reconstruction which is less susceptible to noise. Furthermore, it is capable of reconstructing data from samples with higher emitter densities than classical methods, speeding up the image acquisition process.^[^ ^20^, ^21^, ^22^ ^]^ DL can also be used to directly infer super‐resolution information from diffraction‐limited images,^[^ ^23^ ^]^ and resolution increases up to tenfold have been reported.^[^ ^24^ ^]^

DL is now a routine tool to enhance or interpret super‐resolution microscopy data, reaching an astonishing level of maturity in a very short time.^[^ ^9^ ^]^ There is, however, untapped potential for the use of DL in the automation of super‐resolution imaging, a field that is much less established, but offers huge opportunity to take these imaging methods to the next level of development.

For drug screening and phenotyping of disease, the use of super‐resolution methods offers rich rewards: being able to explore how candidate treatments interact with subcellular machinery at the organelle or even the molecular level is possible with these techniques. However, the need for manual intervention introduces a bottleneck in terms of how many samples can be imaged at a time, and how long a single sample can be imaged for. Their limited throughput makes it impossible to explore a large parameter space, such as when comparing the efficacies between different treatments, or performing dose response studies. The ability for microscopes to perform complex image tasks autonomously would usher in a new era for biomedical research. This topic is particularly exciting, since the entry barriers for use of these advanced technologies are constantly being lowered through the availability of low‐cost and open‐source technologies.^[^ ^25^, ^26^, ^27^, ^28^, ^29^, ^30^ ^]^

In this review, we focus on the most recent applications of DL to achieve automation. We concentrate on three central questions that must be addressed to build autonomous super‐resolution imaging platforms: what to image, when to image, and how to image. We structure our review around these tasks; but first, we begin with a brief summary of current super‐resolution imaging methods.

### 1.1 Super‐Resolution Imaging Methods

There are three key super‐resolution methods: STED,^[^ ^31^ ^]^ SMLM,^[^ ^32^, ^33^ ^]^ and SIM.^[^ ^34^, ^35^ ^]^ While each of these has spawned its own family of derivatives, most super‐resolution techniques rely on one of the core principles. STED is a laser scanning imaging technique based on confocal fluorescence microscopy. Here, a resolution increase is achieved through the depletion of fluorophores around a central excitation spot, yielding a smaller excitation point spread function (PSF) to improve resolution. In SMLM, single fluorophores in the sample are imaged sequentially. This permits their spatial distinction from one another even if their distance is within an area defined by the instrument point PSF, conditions under which they could not be resolved in classical imaging. SMLM requires control over the number of fluorophores that emit detectable photons during image acquisition, and a suitably low on‐to‐off ratio is needed to distinguish proximate emitters. Variants such as stochastic optical reconstruction microscopy (STORM),^[^ ^36^ ^]^ direct STORM (*d*STORM),^[^ ^37^ ^]^ and photoactivation localization microscopy (PALM)^[^ ^38^, ^39^ ^]^ achieve this, either with reversible chemical quenching or photoactivation of fluorophores. A recent variant, pointillistic accumulation in nanoscale topography (PAINT), exploits transient binding between label and target to simulate a switching process. Techniques such as MINFLUX^[^ ^40^ ^]^ microscopy and MINSTED^[^ ^41^ ^]^ combine aspects of STED and SMLM. Finally, SIM projects patterned light onto the sample, generating beats between the structures of the excitation pattern and the object. The resulting low frequency Moiré fringes in the images encode high frequency sample information which can be recovered using Fourier or DL methods.^[^ ^42^ ^]^

The latest developments in these techniques, as well as their relative merits and trade‐offs, have been covered in detail in recent reviews.^[^ ^43^, ^44^, ^45^ ^]^ However, in the context of automation, the use of DL is still in its infancy. Performing super‐resolution microscopy is laborious and requires high levels of user training. Finding objects or phenomena of interest in the sample, tracking these, and maintaining optimal microscope performance requires frequent manual intervention and user skill. The resulting low throughput means automation is crucial to deploy super‐resolution microscopy in complex phenotyping tasks and for experimental scale‐up. It is on these latter topics we focus this review.

## 2 What to Image

**Figure 3:**
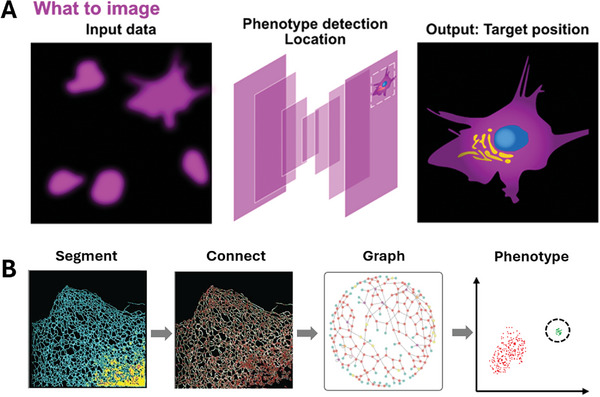
The combination of DL and image‐based phenotyping enables microscopes to take decisions on what to image. A) Whole cell phenotypes. Here, the network decides from low resolution images what to image to maximize the value of the image data. Choosing suitable targets within whole cell populations ensures that rare phenotypes can be captured, without the need to image every cell in the population at super‐resolution. This maximizes efficiency and improves throughput. B) High resolution phenotyping. More complex phenotypes, e.g., relating to subcellular events, can be gleamed from DL analysis of super‐resolution images. Quantitative readouts can be extracted from segmented super‐resolution images of subcellular structures, such as the ER shown here. A subsequent unsupervised graph analysis and clustering of cells by phenotype allow the identification of population outliers. These can then inform the next stages of the imaging process. Adapted from Lu et al. with permission.^[^ ^46^ ^]^ Copyright 2023, Springer Nature.

Reaching machine autonomy in super‐resolution requires the instrument to identify and track features in an image on the fly, and then to take appropriate action. Choosing what to image is at the beginning of this pipeline (**Figure** **3**) and is a formidable task in the context of super‐resolution microscopy. E.g., a researcher may be interested to see how intracellular organelles behave under various treatment conditions, or to monitor those cells in which a specific biomolecular interaction takes place. In classical imaging, this means that cells of interest are identified by the microscope operator, regions of interest are determined, and data acquisition is started. Automating this process is challenging. Agents for automation require an ability to identify relevant subsets from cell populations in which cell‐to‐cell variability may be high^[^ ^47^, ^48^ ^]^ and change over time. Similarly, cells may have to be discriminated from one another in co‐cultures containing multiple cell types (e.g., distinguishing glial cells from neurons). Sample and experimental constraints may mean that it is impossible to image every cell at super‐resolution, and super‐resolution imaging must be targeted to those cells which exhibit the relevant phenotypes (e.g., translation of a specific protein). One step toward automating these tasks is data‐driven microscopy (DDM),^[^ ^49^ ^]^ and the related more recent iteration task‐driven microscopy,^[^ ^50^ ^]^ which use phenotypic information from low‐resolution images to choose regions to be imaged at a higher resolution (Figure 3A). The initial low‐resolution screening provides the user with a broad overview of features, allowing them to choose outliers and cells of interest. This ensures that rare phenotypes can be identified, and that findings can be put in the context of the wider cell population. As well as choosing which cells to image, there is an additional need for a finer control of the imaging area, particularly in super‐resolution imaging where there are unique constraints on the sample. E.g., successful application of SMLM^[^ ^51^ ^]^ requires a field of view (FOV) with a suitable density of labels to permit accurate localization. Similarly, SIM^[^ ^52^ ^]^ and STED^[^ ^53^ ^]^ are both highly sensitive to optical aberrations and out‐of‐focus light. To ensure high fidelity super‐resolution images, FOVs exhibiting these features should be avoided. In choosing what to image, these decisions on the suitability of a FOV for super‐resolution imaging are where the experience of the user is most valuable. Current solutions to this problem revolve around the post‐acquisition screening of acquired data, using either classical or DL analysis to exclude images that are unsuitable for further processing.^[^ ^54^, ^55^ ^]^ This has the obvious disadvantage, however, that the sample is exposed to unnecessary phototoxicity, and time is wasted acquiring unusable data. In the future, tools to determine what to image will need to also incorporate a more refined screening of FOVs.

Identifying those regions in a sample which are suitable for high resolution imaging is one task in the “What to Image” pipeline. Another is to identify those regions that have biological relevance for the process under study. While tools exist to generate datasets of cell phenotypes, the process of choosing which features to focus on is so far still determined by the user. The challenge lies in knowing what phenotypes to look for, especially when some phenotypes are yet to be discovered or could only be observed through super‐resolution imaging. In the most powerful iteration of automated microscopy, the phenotype detection process would be completely unsupervised and make use of DL tools for sophisticated feature extraction^[^ ^56^, ^57^, ^58^, ^59^ ^]^ and fully unsupervised detection of outliers.^[^ ^60^, ^61^ ^]^ For instance, it has recently been demonstrated that super‐resolution imaging of the endoplasmic reticulum (ER) provides a sensitive readout of cellular metabolism.^[^ ^46^ ^]^ Metabolic changes or stress lead to alterations in ER phenotypes, such as dynamic changes in topology. These phenotypes are too subtle for manual quantification, but DL can convert such image data into measurable parameters. Neural networks can automatically segment, skeletonize, and quantify ER network topologies from super‐resolution images of live cells by analyzing parameters such as node connectivity and ER fragmentation. These readouts serve as indicators of diseases such as Niemann‐Pick disease and hereditary spastic paraplegia. There are countless conceivable experiments along these lines using other intracellular readouts for phenotype‐driven microscopy.

**Figure 4:**
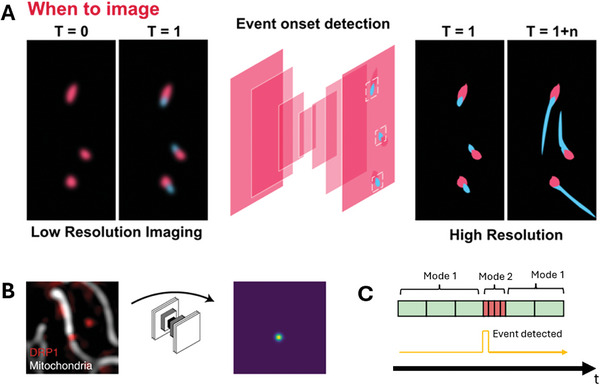
Event‐triggered imaging maximizes data value and preserves sample health. A) Principle of event‐driven imaging. A pre‐determined biological trigger, for example the recruitment of proteins to a point of interest in the cell, switches a change in imaging modality. B) A DL analysis of images permits the detection of complex features inside a cell. The example highlights a mitochondrial fission event, as indicated by the protein DRP1. Adapted from Mahecic et al. with permission.^[^ ^62^ ^]^ Copyright 2022, Springer Nature. C: Operational timeline for event‐driven imaging. The microscope continually images the sample with gentle illumination, minimizing photodamage to the sample during monitoring (‘Mode 1′). When an event is detected, the microscope switches to a higher spatiotemporal resolution modality (‘Mode 2′) for the duration of the event before returning to the initial imaging mode.

## 3 When to Image

Once a FOV has been selected for imaging, either autonomously or by the user, the next question is when to perform imaging. E.g., even though which cell to image might be determined by analysis of its morphology, the precise biological process of relevance may only occur during a division event or at a particular developmental stage. In such cases, it is impractical to image a sample continuously during periods of low activity. In super‐resolution imaging, this is a particularly pertinent issue, since fluorophore bleaching and phototoxicity are usually more pronounced than in conventional fluorescence microscopy.^[^ ^63^, ^64^ ^]^ One approach to mitigate this issue is event‐driven imaging where the microscope adapts the imaging parameters in response to a trigger (**Figure** **4**). By imaging at low photon densities between events, and switching to super‐resolution modes only when an event of interest is detected, the light dose received by the sample is decreased. This extends the duration over which live cells can be imaged without harm and improves the physiological relevance of the measurement. Such an approach has been demonstrated for STED,^[^ ^65^ ^]^ SIM,^[^ ^62^ ^]^ and Bessel‐beam light sheet microscopy.^[^ ^66^ ^]^ In the super‐resolution domain specifically, Alvelid et al. pioneered event‐driven STED microscopy. Here, the microscope switched autonomously between widefield and scanning STED imaging modes.^[^ ^65^ ^]^ As triggers, local intensity changes in widefield images were used to detect calcium spikes in firing neurons, and the onset of exocytosis events in HeLa cells. The fleeting nature of such events means that human users are unable to respond quickly enough to capture them with super‐resolution imaging. The authors were able to activate the STED measurement 40–70 ms after a trigger. This enabled the observation of synaptic vesicle rearrangements following neuronal firing events, and cholesterol dynamics at the point of membrane fusion during exocytosis. While the triggers employed did not require the use of ML, the example demonstrates the power of event‐driven microscopy, surpassing human capability.

An example of event‐driven microscopy enabled by ML was demonstrated by Mahecic et al. The authors performed on‐the‐fly analysis of images with a convolutional neural network (CNN) to predict the onset of division events.^[^ ^62^ ^]^ By training the network on manually annotated video sequences, the authors were able to use the CNN to predict when a mitochondrial or bacterial division event was imminent. They used this to trigger a change in the acquisition speed of an instant‐SIM microscope, allowing observation of the division process at super‐resolution with a reported reduction in photobleaching by almost a factor of five.

Although promising, these techniques have so far remained only semi‐autonomous, requiring prior knowledge of which sample features to monitor. As with decisions on what to image, further development will need to focus on removing the requirement to define specific cues. This could be achieved, e.g., by monitoring more generic time‐asymmetric processes,^[^ ^67^ ^]^ or by adopting an unsupervised approach to event detection.^[^ ^61^, ^68^ ^]^ Bouchard et al. have made some progress toward this goal by using single‐image super‐resolution reconstructions generated from widefield images as triggers.^[^ ^69^ ^]^ By analyzing regions of high frame‐to‐frame variability in a time series of such images, they were able to predict where biological processes were occurring below the diffraction limit by looking for regions where the network was unsure about the structure. This enabled the observation of rare events involving the reorganization of F‐actin in neural dendrites, whilst avoiding the need for continuous super‐resolution imaging. While the single‐image super‐resolution method they employed was tailored to the reconstruction of actin structures, developing more generalized networks compatible with a wider range of structures would allow for more flexible event triggering. Compared to methods where DL is only used to improve image resolution, this approach also has the further advantage that findings are validated through true super‐resolution, increasing user faith in the data.

## 4 How to Image

**Figure 5:**
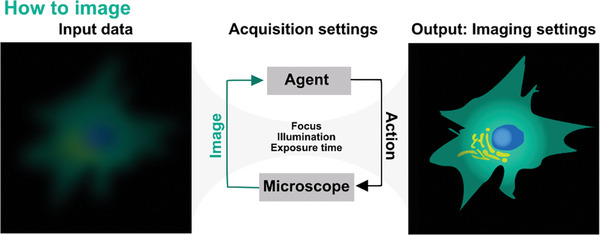
Analysis of image data from the microscope allows real‐time optimization of imaging parameters. An ML agent acts in a feedback loop where features extracted from the image data with DL are used to predict which hardware function need adapting (e.g., focus position, illumination power, etc.). After instrument parameters have been updated and new images acquired, the loop is repeated. In this way, the agent achieves continuous optimization during imaging.

The final decision that must be made in the imaging process is how to image, that is choosing the imaging parameters (e.g., exposure time, illumination intensity, etc.) or even the imaging modality (**Figure** **5**). The automation of single microscope operations with DL is rapidly increasing in popularity. Examples include sample focusing^[^ ^70^ ^]^ and aberration estimation in adaptive optics. For SIM, DL aberration estimation has enabled super‐resolution imaging of the neuromuscular junction deep inside developing *Drosophilla* larvae, where the presence of out‐of‐focus light hinders classical Fourier‐based aberration estimation methods.^[^ ^71^ ^]^

While single‐purpose DL tools such as these can assist a user, automating individual functions is not sufficient for truly autonomous imaging, which requires the simultaneous optimization of multiple parameters. This is particularly true when the choice of parameters requires a balance of conflicting outcomes, such as the trade‐off between spatiotemporal resolution and photodamage. Reinforcement learning (RL)^[^ ^72^ ^]^ offers one possible solution to this automation problem. In RL, ML “agents” are trained to choose an action based on the current state of an environment in order to maximize some reward. As an ML technique, RL is particularly well‐suited to the operation of systems with multiple controls, where choosing the best action may be a compromise between different outcomes. For this reason, RL has found use in a wide range of tasks from spacecraft control and route planning to astronomical imaging.^[^ ^75^, ^76^, ^77^, ^78^ ^]^ In microscopy, RL has been used to choose optimal illumination schemes for cell classification in Raman microscopy,^[^ ^73^ ^]^ and for aberration correction in widefield microscopy.^[^ ^74^ ^]^

In super‐resolution microscopy, Durand et al. demonstrated online training with RL to automatically optimize excitation power, depletion power and pixel dwell time in a STED microscope. For this online approach, the agent was trained during a brief ‘exploration’ phase, where it could learn, through trial‐and‐error, which parameters impacted image quality on a particular sample.^[^ ^75^ ^]^ In a further development, they demonstrated that training could be guided by either expert feedback or in a fully autonomous fashion, where a pre‐trained DL network was used to assess image quality. While this online approach is effective, the need to train the agent on a sample‐by‐sample basis means that this method is not suitable for the imaging of fragile samples or those in which the regions of interest may be very sparse. One way to mitigate this problem is to train RL agents in a simulated environment, allowing the agents to learn on a diverse dataset, generalizing to perform on any sample. In microscopy, one such simulation environment for is PySTED, which simulates STED images based on microscope and sample parameters.^[^ ^76^, ^77^ ^]^ To provide the wide range of sample types needed, Bilodeau et al. developed a DL technique to approximate the ground truth distribution of fluorophores from STED images. This allows PySTED to make use of both a priori knowledge of the photo‐physical behavior of fluorophores, and their biologically relevant spatial distributions. In their implementation, PySTED has been used to generate synthetic data for cell segmentation tasks, and to train agents to control excitation power, depletion beam power, dwell time, and pixel size. Importantly, this training approach requires no retraining of the agent before deployment to the microscope.

## 5 Perspectives and Conclusions

### 5.1 Advances in DL for Automation

One of the most profound advances in ML has been the recent development of large language models (LLMs). At their heart, LLMs are neural networks trained on large datasets for sequence processing.^[^ ^78^, ^90^ ^]^ The most familiar example of such a task is predicting the next word in a text string based on an input sequence of text in the form of a written prompt, an ability that is increasingly being used in academic publishing.^[^ ^79^ ^]^ More generally, however, LLMs hold extraordinary potential for any task that can be framed as sequence prediction. In biology, one such example is the generation of proteins from sequences of amino acids, a capability recently demonstrated by Mandi et al.^[^ ^80^ ^]^ Image processing tasks can similarly be achieved with LLMs by considering an image as a sequence of image patches or tokens.^[^ ^81^ ^]^ Furthermore, by encoding different data types into tokens, multi‐modal LLMs can simultaneously interpret different data formats (e.g., images and text).^[^ ^82^ ^]^ In image analysis, several such multi‐modal LLMs have been proposed as potential diagnostic tools in medical imaging.^[^ ^83^, ^84^, ^85^, ^86^ ^]^ However, currently these generally underperform against CNNs and require extensive prompt‐engineering to minimize hallucination.^[^ ^87^ ^]^ Multi‐modal LLMs have also been tested in microscopy, for image processing and classification tasks. However, all the available LLMs performed worse than human experts and, at times, simply refused to perform a task.^[^ ^88^ ^]^

Combined with their ability to interpret the meaning of user requests, multi‐modal LLMs also allow for semantic hardware control.^[^ ^89^ ^]^ These principles have been demonstrated in experiments, such as reported by Liang et al., where LLMs for code generation were used to write and execute code for the control of a robotic system in response to user requests.^[^ ^90^ ^]^ In the context of microscopy, this abstract interpretation of meaning would allow users to make objective‐oriented requests (e.g., “Image cells undergoing apoptosis”) rather than needing to specify what features to look for (e.g., “Image cells with high eccentricity”). Even without multi‐modal capabilities, collaborative collections of multiple LLMs, such as Coscientist,^[^ ^91^ ^]^ are now capable of designing new experimental workflows based on a research question, and then autonomously perform the experiments whilst simultaneously performing error‐detection and correction. Integrating automated microscopes into such LLM‐generated workflows would greatly expand the capabilities of automated discovery. In a more supervised fashion, this level of planning could offer users guidance on which imaging modality to use based on their specific research question.^[^ ^45^ ^]^

So far, almost all LLMs have benefitted from the scaling of the computational resources used during training, i.e., by assembling progressively larger datasets to train bigger models. An alternative is scaling the resources used during inference, to dedicate more computations to more complex problems.^[^ ^92^ ^]^ An example of this is the chain‐of‐thought prompting of LLMs, where LLMs produce outputs through sequential reasoning tasks. Most recently, GPT‐o1 was trained specifically with this capacity in mind, which resulted in significant performance gains over alternative models.^[^ ^93^ ^]^ The intermediate reasoning steps achieved with chain‐of‐thought prompting can be further augmented by granting the model the ability to collect additional data.^[^ ^94^, ^95^ ^]^

In addition to LLMs, Graph neural networks (GNNs)^[^ ^96^ ^]^ offer great promise for application in automated super‐resolution microscopy. GNNs were developed for the processing of data where connectivity is an important feature. This makes GNNs naturally suited for tasks where tools developed for graph analysis are commonly used. This includes the study of particle tracking^[^ ^97^, ^98^ ^]^; the morphology of organelles with network‐like structures (such as ER)^[^ ^46^ ^]^; and inter‐cellular connectivity (of relevance to efforts to map entire neural networks^[^ ^99^ ^]^). In the future, multi‐modal GNNs,^[^ ^100^ ^]^ capable of incorporating data from different domains would not only provide an efficient representation of imaging data, but could also incorporate data on, e.g., treatment conditions or neuronal activity.^[^ ^101^, ^102^ ^]^ When used as tools for autonomously deciding what to image, such multi‐modal GNNs would allow for the investigation of more complex dependencies between morphological features and disease.

### 5.2 Expanding the Scope of Hardware Automation

As well as improvements to the underlying DL techniques, furthering the capabilities of automated super‐resolution microscopy will also require automation of a wider scope of experimental parameters. To this end, there have been some efforts to integrate automation of the sample handling process into imaging.^[^ ^103^, ^104^, ^105^ ^]^ E.g., by incorporating microfluidics into a super‐resolution microscope, it is possible to automate buffer exchange in DNA‐PAINT imaging.^[^ ^104^, ^106^ ^]^ Combined with techniques for multiplexing acquisition, this would allow for far more targets to be imaged in one experiment than could be achieved by a human operator.^[^ ^107^, ^108^ ^]^ In the future, such tools could also allow the microscope to choose which structures to label based on the user‐defined research question, or a provisional, artificially labeled image.^[^ ^109^, ^110^, ^111^ ^]^ Alternatively, by combining sample environment control with techniques for image‐based monitoring of cell health,^[^ ^46^ ^]^ the microscope could adjust reagent concentrations in response to changes in phenotype, or begin sample fixation when cells show signs of phototoxic morphology changes. These approaches would ensure that image data represent the natural, or physiological, cell state. Similarly, the ability to control the environment of the sample could be exploited to discover novel phenotypes. Here, curiosity‐driven reinforcement learning models specifically trained to generate novel observations hold particular promise.^[^ ^112^ ^]^ While curiosity‐driven RL currently requires – in the context of super‐resolution microscopy – an infeasible amount of training data, more recent algorithms significantly reduce the size of datasets required by enabling fast training on real‐world data.^[^ ^113^, ^114^ ^]^ At the same time, the automation of the sample handling and imaging process enables a much higher rate of data collection, potentially making curiosity‐driven RL a more feasible approach as both technologies mature.

### 5.3 Generating Training Data Suitable for Automated Imaging

While there have been great strides in ML, transferring these developments to microscopy, and super‐resolution microscopy in particular, is challenging. One of the key hurdles to overcome is the availability of suitable data for training.^[^ ^115^ ^]^ It is vital during training that DL networks are provided with sufficiently large datasets so as to generalize to a task (i.e., successfully operate on varied data during inference).^[^ ^116^ ^]^ At the same time, it is important to minimize the effects of dataset drift where differences between the distributions of the training data and inference data lead to erroneous outputs.^[^ ^117^ ^]^ This requires not only a high quantity of training data, but also a high quality. In super‐resolution microscopy, obtaining such datasets in the real world is challenging due to the limitations in throughput and the heterogeneity of both instrumentation and samples. To help address this, there is a growing trend in the imaging community for data sharing, and datasets for a range of imaging techniques are now available.^[^ ^115^ ^]^ An alternative solution to this problem is to use transfer learning where ML networks are trained not on microscopy data, but on synthetic data generated either from scratch or from more expansive natural image libraries; a technique which has proven effective in the development of reconstruction algorithms for SMLM and SIM.^[^ ^17^, ^21^, ^118^ ^]^

A final approach is to use DL techniques themselves to generate artificial datasets. Generative adversarial networks (GANs)^[^ ^119^ ^]^ have been shown to be capable of creating suitable training data, both unconditionally (i.e., from pure noise), and by converting existing data from one imaging modality to another.^[^ ^120^, ^121^, ^122^ ^]^ More recently, diffusion models^[^ ^123^, ^124^ ^]^ have become widely adopted as an alternative method for image generation, including the generation of images from a text prompt. This works by encoding text into an abstract latent representation, as first demonstrated with contrastive language‐image pretraining (CLIP).^[^ ^125^, ^126^ ^]^ In principle, such a representation could instead come from any input modality, including a representation of organelle morphology or network connectivity – encodable with other neural networks. E.g., artificial ER samples could be generated by modifying the representation of an existing ER structure by a GNN, potentially resulting in more representative samples than allowed for by other methods. For more complex sample simulations, neural radiance field models (NeRF)^[^ ^127^ ^]^ have enabled the generation of 3D objects,^[^ ^128^, ^129^, ^130^ ^]^ which may provide a better representation of biological samples. Similarly to the above, these models can generate outputs based on a latent representation of data from a different modality such as text.^[^ ^131^ ^]^ Finally, neural cellular automata^[^ ^132^ ^]^ can enable data generation in both 2D and 3D, as well as its evolution in time. Here, a neural network can learn a local update rule for a cellular automaton^[^ ^133^ ^]^ to generate highly complex patterns the evolve with time. These could potentially enable the generation of synthetic data, that mimics both the structures and dynamics of in vivo microscopy training data.

### 5.4 Improving User Uptake

Finally, and perhaps most importantly, key to the future widespread use of ML for automation is user uptake. Currently, there are two major factors holding users back: user faith in automation tools and their accessibility. Since the beginning of their widespread use, ML networks have consistently been viewed as “black‐box” algorithms, where it is often not possible to determine why a network gave a particular output or chose to take a particular action. For the automation of super‐resolution microscopy, this lack of transparency raises two issues. First, if the ML agent made an incorrect decision, it is difficult to diagnose what the cause was, and how to tune the network to prevent the same error from occurring again. Second, it is challenging to determine if the agent has unintentionally learned a bias from the training data. In the context of choosing what to image, such a bias could result in phenotypes being missed or over‐represented in later statistical analysis. Because of these limitations, the field of explainable AI (XAI) is increasingly attracting attention.^[^ ^134^ ^]^ The goal of XAI is to develop frameworks where researchers have access to the mechanisms by which model outputs are produced. In biomedical research involving patient‐oriented data, on which diagnoses are made or therapies designed, this is crucial and XAI tools have been reviewed comprehensively.^[^ ^135^ ^]^ Similar attempts have been made to open the black box of LLMs. Retrieval‐augmented generation, where the model is given the ability to access data from an external database, is one potential approach as it not only makes outputs more reliable, but also improves interpretability.^[^ ^136^, ^137^ ^]^ At the same time, research into interpretability has made it possible to identify the function of individual parameters of LLMs, or to find groups of parameters that correspond to a single concept or behavior.^[^ ^138^ ^]^ Finally, while chain‐of‐thought prompting emerged as a way of forcing the models to exhibit a form of more complex reasoning, it can also enable the user to follow the logic employed by the ML agent. In the future, incorporating these tools into ML for super‐resolution microscopy will likely increase the uptake of the methods by increasing faith in the technology.^[^ ^139^ ^]^

To improve the accessibility of automated super‐resolution microscopy, there is a growing toolbox of software available to researchers. One of the first of these was Micropilot,^[^ ^140^ ^]^ built around LabVIEW and C which enabled the real‐time control of microscope hardware in response to image data. More recent examples of automation tools include AutoscanJ^[^ ^141^ ^]^ – which allows users to define events or structures of interest through ImageJ scripts – and CyberSco.Py^[^ ^105^ ^]^ and MicroMater^[^ ^142^ ^]^ which operate through Python. Vital in this field has been the drive toward open‐source software for hardware control.^[^ ^143^, ^144^ ^]^ The most notable example is micromanager, which is specifically designed to interface with a range of hardware from different manufacturers. This greatly facilitates the development of custom‐built microscopy setups and, with libraries such as Pycro‐Manager,^[^ ^145^ ^]^ offers users the lower‐level programmatic control needed to control the how to image.

## 6 Conclusion

We are witnessing the beginning of the ML revolution in microscopy. Among its many applications, we believe the automation of the super‐resolution imaging process holds the greatest potential. By eliminating the need for researchers to supervise the experiment and enabling the detection of even the most subtle phenotypes, this new technology could transform biological research and drug discovery.

## Conflict of Interest

The authors declare no conflict of interest.
